# Sex-specific energy expenditure during the Alaska mountain wilderness ski classic; insights from an Arctic winter expedition

**DOI:** 10.3389/fphys.2025.1543834

**Published:** 2025-04-29

**Authors:** Melynda S. Coker, Michelle N. Ravelli, Timothy C. Shriver, Dale A. Schoeller, Dustin R. Slivka, Brent C. Ruby, Robert H. Coker

**Affiliations:** ^1^ Montana Center for Work Physiology and Exercise Metabolism, School of Integrative Physiology and Athletic Training, College of Health, University of Montana, Missoula, MT, United States; ^2^ Isotope Ratio Mass Spectrometry Core Laboratory, University of Wisconsin, Madison, WI, United States

**Keywords:** resilience, cold, physical activity, survival, human

## Abstract

**Purpose:**

The Alaska Mountain Wilderness Ski Classic (AMWSC) is a self-supported and self-oriented winter expedition that occurs in the remote North American Brooks Range, ∼200 km north of the Arctic Circle. Few investigations have evaluated sex-specific physiological responses under extreme cold and isolated circumstances. Our study examined sex-specific differences in total energy expenditure (TEE), water turnover (WT), and changes in body composition during the expedition.

**Methods:**

Twenty adult participants (8 females, age: 41 ± 6 years, body mass index: 22.8 ± 1.9 kg/m^2^ and 12 males, age: 38 ± 4 years, body mass index: 22.7 ± 1.6 kg/m^2^) enrolled in and completed the study. TEE and WT were examined during the expedition using the doubly labeled water (DLW) method. Body composition was measured using multi-frequency bioelectrical impedance.

**Results:**

The duration of the expedition was similar in females (8.1 ± 1.6 days) and males (7.5 ± 0.9 days). Absolute rates of TEE were lower in females (20.8 ± 4.7 MJ/day) compared to males (31.1 ± 7.5 MJ/day). However, when expressed relative to fat free mass (FFM), rates of TEE were similar in females (0.42 ± 0.07 MJ/FFM/day) and males (0.45 ± 0.10 MJ/FFM/day). TEE/body mass plus pack weight (i.e., total load carriage) was lower in females compared to males. WT was reduced compared to previous reports of athletes exercising in thermoneutral and hot environments.

**Conclusion:**

Absolute rates of TEE were lower in females compared to males, but there was no difference when TEE was expressed relative to fat free mass. Estimates of TEE/total load carriage were lower in females than males, modestly suggesting greater functional efficiency in females during this expedition. Compared to other ultra-endurance events in warm environments, WT may have been reduced by lack of water availability, self-selected reductions in exercise intensity, and limited sweat loss.

## 1 Introduction

Human-powered movements during the winter in Arctic alpine regions are complicated by interactive relationships between extreme cold, up to 24 h of daily darkness, isolation from existing infrastructure, and the need for land navigation and mountaineering skills (Melvin et al., 2011; Strawa et al., 2020). These anomalies present physiological stressors that require expertise, patience, agility, and intuition for survival, even without the demands of physical activity. In an emergent scenario, air support or air extraction can be extremely dangerous, dependent upon multiple factors (i.e., visibility, weather patterns, snow depth and quality, distance from civilization, etc.), therefore limiting the feasibility of rescue and/or resupply (Haagensen et al., 2004). Thus, the inextricable links between these factors and physical injury, equipment failure, and communication breakdown in this austere environment can have devastating consequences, leading to an increased risk of mortality (Kadir et al., 2013).

To maximize physiological resilience and adequate preparation in these and other extreme circumstances, pragmatic clinical studies are desperately needed (Winter, 2022; Conkright et al., 2022). Little is known about the sustainment of human-powered movement under such conditions, especially in females (Ahmed et al., 2020). Estimates of total energy expenditure (TEE) provide a foundational perspective for proper training and nutrient delivery strategies in Arctic winter conditions (Ahmed et al., 2020). TEE may increase simply due to the requirement for bulky and heavy personal protective equipment (Ahmed et al., 2020; Johannsen et al., 2018) and a seasonal change in basal metabolic rate (BMR) (Leonard et al., 2002). Snow, ice, and difficult terrain complicate mobility and require energy demand, especially when challenged by load carriage (Johannsen et al., 2018). In these situations, it is unknown whether sex-specific variations in morphology and body composition promote differences in TEE (Johannsen et al., 2018).

In addition to the importance of nutrient consumption in an Arctic winter environment, water intake is a vital consideration (Popkin et al., 2010). While it is well known that body water is lost in urine, sweat, feces, and respiratory water vapor, sufficient water intake can be challenging during almost constant physical activity (Popkin et al., 2010). This challenge is exacerbated by sustained sub-zero temperatures that complicate the procurement of water. We anticipate that the merger of these factors will reduce water intake. To our knowledge, water turnover (WT), which reflects both water intake and loss, has not been investigated in the context of athletes expending substantial TEE in an unscripted Arctic winter environment.

We previously estimated energy expenditure using GT3x + Actigraph accelerometers and ActiLife software in female and male individuals participating in the Alaska Mountain Wilderness Ski Classic (AMWSC). This event is an unsupported, point-to-point, trailless, and remote expedition in which participants traverse remote mountain ranges in Alaska under Arctic winter conditions (Johannsen et al., 2018). We described higher Actigraph-derived energy expenditure in males than females, which was consistent with 36% higher lean body mass in males. Using data derived from these wearable devices and dual-energy x-absorptiometry scanners, estimates of energy expenditure/lean body mass were relatively similar between females and males. Females carried more pack weight per kg of body mass than males (Johannsen et al., 2018). However, the estimation of TEE using wearable biometrics in this cohort should be evaluated with caution because the algorithms used to translate vector magnitudes are not specific to cross-country skiing (Trost et al., 2005). Some military training exercises have suggested similar findings with regard to TEE expressed per kg of body mass and fat-free mass (Castellani et al., 2006; Hoyt et al., 2006), but these studies were not performed under sustained sub-zero Arctic winter conditions.

The objective of the current study was to determine TEE and WT in females and males participating in the remote, unscripted AMWSC expedition using the doubly labeled water (DLW) method. We hypothesized that TEE expressed per FFM would be similar in females and males despite expected differences in absolute TEE (MJ/day). We also sought to evaluate WT in our study compared to other ultramarathon events in thermoneutral or hot conditions.

## 2 Materials and methods

### 2.1 Study design and participants

**FIGURE 1:**
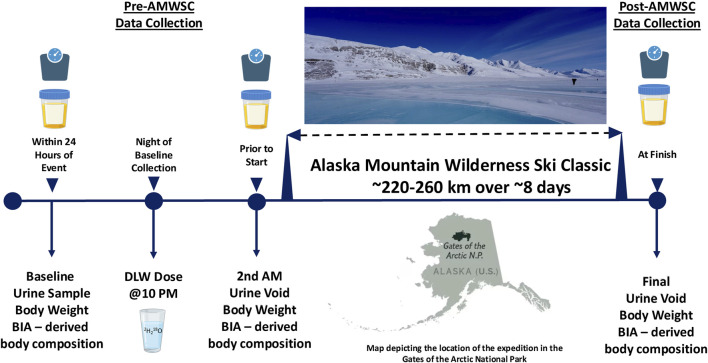
Determination of total energy expenditure during the Alaska mountain wilderness ski classic.

We recruited 12 men (age: 38 ± 4 years) and nine women (age: 41 ± 6 years) from individuals participating in the AMWSC. One day prior to the expedition, participants arrived at the Arctic Getaway Bed and Breakfast (AGBB) located at 8 Igloo St, Wiseman, AK 99701. Formerly a small mining community, Wiseman has a full-time resident population of 12 individuals and was only recently connected to the road system (i.e., Dalton Highway) in the early 1900s. There, we obtained written consent, provided instructions, and performed all aspects of data collection (Figure 1). Documents for consent were provided to participants in written form and then verbally described. Following opportunities for questions, a signed consent was obtained for each participant. Upon completion of the consent process, a comprehensive health history questionnaire was utilized to ensure that all participants were healthy, non-smoking, and had no chronic inflammatory conditions. No participants were taking any medications, nor were they symptomatic for cardiovascular, respiratory, neurological, or metabolic diseases. Also determined were (a) nude body mass, (b) body composition, and (c) urine samples were obtained (listed below in further detail). Post-AMWSC data collection return visits to the AGBB were completed by each participant upon the conclusion of the expedition. All aspects of the study and related documentation were reviewed and approved by the University of Montana (UM) Institutional Review Board (IRB) and the Air Force Human Research Protections Office (HRPO).

### 2.2 Alaska mountain wilderness ski classic

*Unlike many other ultra-endurance events or competitions, the AMWSC is not a race*. It is not a timed competition. There are no trophies or awards. Rather, it is a unique, extreme wilderness expedition that emphasizes unequivocal self-reliance, backcountry skiing, mountaineering experience, and a zero trace–zero impact philosophy in an unscripted Arctic alpine winter environment. There are no trails. There are no aid stations. Athletes must rely on their own land navigation skills to select routes of passage based on weather conditions, avalanche risks, topography, overflow ice, food supply, physical ability, and equipment selection for themselves and their “teammates.” As a result, routes can change day by day or even hour by hour. Athletes self-selected high quality technical clothing that included wool underwear, breathable pants and tops, and synthetic and/or down insulated clothing with or without a waterproof shell. The AMWSC captures the essence and difficulties of unscripted human-powered movement in austere circumstances characterized by the harsh conditions of the Arctic winter.

All testing and examinations of the research participants for the pre-and post-AMWSC were completed on location at the AGBB in Wiseman, Alaska, during April of 2023 and 2024. These sessions were closely coordinated with AMWSC expedition organizers, minimizing the logistical and physiological burden on the participants. This collaborative partnership allowed us to complete testing procedures within 24 h of the start and 4 h of completion of the AMWSC. Weather conditions were derived by the National Weather Service measurements at Anaktuvuk Pass (68º08′35´´N 151º44′01´´ W), which is located slightly north of the Brooks Range and between the Anaktuvuk and John Rivers. These measurements provided conservative yet highly accurate data on the weather conditions during the AMWSC (Table 1).

**TABLE 1:** Average climate conditions during the 2023 and 2024 AMWSC.

Time of Day	Low Temp (°C)	High Temp (°C)	Wind Speed (mph)	Wind Chill Low Temp (°C)	Wind Chill High Temp (°C)	Irradiance - Low Temp (Watts/M^2^)	Irradiance – High Temp (Watts/M^2^)
0600 HR	−24.8	−22.2	9.1	−33.8	−31.1	1732	1575
1200 HR	−24.2	−21.1	9.5	−29.8	−29.8	1731	1635
1800 HR	−20.3	−18.5	10.1	−29.1	−26.9	1635	1598
2400 HR	−23.2	−19.0	10.7	−33.4	−28.1	1769	1636

### 2.3 Body mass and body composition

Within 24 h of the start of the AMWSC expedition, participants were given a private area at the AGBB and instructed to disrobe and step onto the portable, dual-frequency full-body body composition analyzer (Tanita DC-430U, Tokyo, Japan). Per specifications from the manufacturer, the measurement range and standard deviation range for impedance is 1,000 ± 2%. After at least 4 h of rest, participants were asked to positionally stabilize themselves on the scale. They were instructed to avoid allowing their inner thighs to touch, to clean the soles of their feet well, and limit movement. Participants held the scale handles with maximum hand-to-surface contact. Arms and hands were placed by their sides until all digital readings were complete. Pre- and post-expedition weight and body composition measurements (i.e., fat-free mass (FFM), fat mass (FM), and body fat percentage) of the participants were then recorded. Each participant returned to the AGBB immediately upon completion of the AMWSC. The same procedures were then repeated to gather post-AMWSC measurements.

### 2.4 Pack weight

The expedition backpacks used by the athletes were weighed before and immediately following the AMWSC (Tanita DC-430U, Tokyo, Japan). Based on the understanding among athletes that food intake was regimented, thus similar each day and solely responsible for reductions in pack weight, we estimated the daily pack weight over the course of the expedition. We then used these data to derive daily TEE relative to body mass plus pack weight, or TEE/total load carriage.

### 2.5 Isotopic methodology

The DLW method was used to measure CO_2_ production, which was then used to calculate TEE (MJ/day). Elimination rates of ^2^H_2_O were used for the determination of WT. Participants were verbally instructed on the protocol requirements, furnished with a timeline diagram detailing each step of their data collection points, and provided with written instructions for all expectations throughout the study. A baseline urine sample was collected ∼24 h prior to the AMWSC expedition to determine background isotopic enrichments. All participants then received an oral dose of DLW based on their body masses (2.52 g·kg^−1^ estimated total body water (TBW)^−1^ H_2_^18^O at 10 atom percent excess, 0.18 g·kg^−1^ estimated TBW^−1 2^H_2_O at 99.8 APE), (Cambridge Isotope Laboratories, Andover, MA, Isotec, Inc., Miamisburg, OH) at ∼22:00 h prior to the AMWSC expedition (Schoeller et al., 1986). Each isotope vial was rinsed three times with ∼30 mL of water, and that rinse was consumed to ensure complete isotope delivery. Participants voided into sterile urine collection cups, and then the samples were transferred into sterile cryovials (Corning, Inc., Corning NY) using sterile disposable pipettes (Uline, Allentown, PA). Urine samples for analysis consisted of the background sample, the second void after dosing (8 ± 2 h post-dosing), and the final void upon completion of the expedition. Cryovials were wrapped in Parafilm™ (Bernis NA, Nina, WI) to prevent evaporation, as previously described (Coker et al., 2018).

Analysis of isotopic enrichments and calculation of TEE were conducted at the Isotope Ratio Mass Spectrometry Core Laboratory at the University of Wisconsin, Madison, as previously described (Schoeller et al., 1986; Hoyt et al., 1991; Ruby et al., 2002). Isotopic abundances were measured by isotope ratio mass spectrometry (IRMS) through equilibration with CO_2_ for oxygen isotopic ratio (^18^O/^16^O) and chromium reduction for hydrogen isotopic ratio (^2^H/^1^H) (Schoeller et al., 1986). Background shifts in enrichment were adjusted as previously indicated (Schoeller et al., 1986).

Dry carbon was utilized to clean urine samples, thus clean fluids were reduced over the chromium column at 850°C. A Finnigan MAT Delta Plus isotope ratio mass spectrometer (Thermo Finnigan, San Jose, CA) was used to conduct the analysis of deuterium abundance (Thorsen et al., 2011). To measure the ^18^O/^16^O ratio, the second urine sample was equilibrated with 1 mL of STP of CO_2_ using a Thermo Scientific GasBench interfaced to a Delta V Plus IRMS (Thermo Scientific Inc., Waltham, MA). Analysis was performed in duplicates with an analytical precision of 0.15% for ^18^O and 0.5% for ^2^H. All samples for each participant were processed within a single batch to ensure consistency. Post-dose enrichments were calculated relative to the baseline abundance. Total CO_2_ production was derived by the turnover rates (*k*) of isotopes of oxygen-18 (*k*_o_) and deuterium oxide (*k*_d_) along with their respective isotope dilution spaces N_o_ and N_d_ (Speakman et al., 2021). The total CO_2_ production was converted to TEE using the Weir equation, assuming a respiratory exchange ratio of 0.86 (Weir, 1949).

WT was estimated by using deuterium dilution space (N_d_) and its elimination rate (*k*_d_), applying the formula: rH_2_O = N_d_ x *k*_d_ (Schoeller et al., 1980). TBW was determined as the average of the N_o_ and N_d_, with a correction for *in vivo* isotopic exchange using a standard adjustment equation: TBW = [(N_o_/1.007+(N_d_/1.043)]/2 (Schoeller et al., 1980).

In addition to the body composition data provided by bioelectrical impedance technology, measurements of FFM and BMR were also derived from the estimation of TBW and a hydration constant for lean tissue of 0.732 (Wang et al., 1999; FAO/WHO/UNU, 2004). The energy expenditure of physical activity (EEA) was calculated as (TEE/BMR) (Westerterp et al., 1986). All analyses were performed in duplicate, and all urine specimens from each participant were analyzed during the same batch.

### 2.6 Statistical analysis

Data are reported as means ± standard deviations (mean ± SD). An F-Test was utilized to assess whether variances existed between females and males. For all variables except WT, where an unanticipated variance was noted, we determined sex-specific differences between females and males using a paired t-test equal variance. For the determination of sex-specific differences in WT, we utilized a paired t-test unequal variance. We also completed a repeated measures analysis of variance whereby the average TEE/body weight plus pack weight, including daily average reductions, were compared between females and males participating in the expedition. We used a conservative multiple comparison Bonferroni for each paired data point in this regard. We imputed missing post-event body composition data for Female 2 due to an equipment malfunction. We imputed data for Male 10 due to an emergent extraction from the field which precluded the measurement of post-pack weight and post-body composition. We were able to acquire all other data from this participant. Statistics were considered significant with a *p*‐value of less than 0.05.

## 3 Results

### 3.1 Climate conditions

The temperature conditions ranged from a low of −36°C to a high of −6°C, respectively (Table 1). Wind speed ranged from five kph to 34 kph (Table 1). Wind chill ranged from −49°C to −11°C (Table 1). Irradiance, or radiant energy flux across a given surface, ranged from 1104 W/m^2^ to 2071 W/m^2^ (Table 1). There were no significant differences in these parameters for 2023 and 2024. Based on the information provided by the expedition organizer, the estimated distance of the 2023 and 2024 AMWSC varied from 220 to 260 km, depending upon the route selected by the participants.

### 3.2 Age, body weight, and body composition

We originally recruited twenty-one adult participants. One of the original nine females recruited dropped out at 1.5 days due to a foot injury. Due to the injury and limited participation, her data was not included in our analysis, so eight females actually completed the study. There were no differences in age or BMI between females and males (Table 2). Pre- and post-body weight and FFM were lower in females compared to males (Table 2). Pre- and post-fat mass and percent body fat were higher in females compared to males (Table 2). There were no significant changes in these variables over the course of the expedition in females or males. Also, there were no differences in pre-expedition FFM derived from TBW compared to pre-expedition FFM derived from multi-frequency bioelectrical impedance in females (*p* = 0.65) or males (*p* = 0.15). The number of days in the field was not different (*p* = 0.9448) between females and males (Table 3).

**TABLE 2:** Body composition Female, male, and combined data are presented as mean ± standard deviation.

Body Composition	Age (years)	Pre-Body Weight (kg)	Post-Body Weight (kg)	Pre-Pack Weight (kg)	Post-Pack Weight (kg)	Pre-Body Mass Index (kg/m^2^)	Post-Body Mass Index (kg/m^2^)	Pre-Fat Free Mass (kg)	Post-Fat Free Mass (kg)	Pre-Fat Mass (kg)	Post-Fat Mass (kg)	Pre-Body Fat (%)	Post-Body Fat (%)
*Female 1*	38	63.3	61.9	21.0	14.0	21.9	21.4	47.7	51.3	15.6	10.7	24.6	17.3
*Female 2*	49	62.6	61.3	24.0	17.0	23.6	23.2	46.3	x	16.1	x	25.8	x
*Female 3*	38	66.1	66.2	21.4	13.9	20.9	20.9	59.4	62.7	6.7	3.5	10.1	5.3
*Female 4*	39	64.4	61.4	21.0	14.5	22.1	21.2	50.8	49.1	13.5	12.3	21.1	20.1
*Female 5*	35	69.8	70.2	24.5	17.3	23.4	23.4	52.8	53.8	17.0	16.4	24.3	23.4
*Female 6*	50	61.6	61.7	18.5	16.2	22.6	22.6	44.5	49.8	17.1	11.9	27.7	19.3
*Female 7*	43	85.1	83.5	17.0	14.0	26.8	26.4	62.8	58.4	22.1	25.1	26.1	30.0
*Female 8*	35	58.9	60.8	22.7	14.0	21.6	22.3	46.5	42.2	12.4	11.3	21.1	18.6
***All Females***	**41 ± 6**	**66.5 ± 7.7**	**65.9 ± 7.3**	**21.3 ± 2.6**	**14.9 ± 1.3**	**22.9 ± 1.7**	**22.7 ± 1.6**	**51.4 ± 6.2**	**52.5 ± 6.2**	**15.1 ± 4.2**	**13.0 ± 6.1**	**22.6 ± 5.2**	**19.1 ± 6.9**
*Male 1*	41	81.2	78.5	23.0	15.0	25.0	24.1	75.0	76.1	6.2	2.4	7.6	3.0
*Male 2*	34	71.5	70.3	23.1	18.1	21.4	21.0	67.1	67.6	4.4	2.7	6.1	3.9
*Male 3*	34	70.7	68.6	23.0	16.1	21.1	20.5	64.8	65.9	5.9	2.7	8.3	4.0
*Male 4*	37	72.0	70.6	21.2	17.0	21.0	20.5	69.6	68.5	2.4	2.1	3.4	3.0
*Male 5*	35	83.2	82.5	21.8	14.5	24.8	24.6	72.5	75.2	10.7	7.3	12.9	8.8
*Male 6*	35	85.5	82.6	20.4	14.7	24.8	24.6	74.5	72.8	10.9	9.8	12.8	11.5
*Male 7*	35	73.0	72.1	25.0	22.2	22.4	22.1	63.1	67.6	9.9	4.5	13.6	6.3
*Male 8*	47	86.8	84.5	20.8	14.3	24.5	23.9	80.4	79.0	6.4	5.5	7.4	6.5
*Male 9*	35	87.4	85.7	22.0	15.1	22.8	22.4	80.5	80.5	6.9	5.5	7.9	6.4
*Male 10*	35	73.5	82.0	22.1	16.2	21.9	24.0	67.9	79.0	5.5	8.0	7.5	8.0
*Male 11*	46	74.1	72.3	20.8	16.5	22.7	22.2	67.4	66.6	6.7	4.1	9.0	7.7
*Male 12*	38	70.4	69.2	24.0	14.8	20.4	20.1	66.8	65.7	3.5	3.5	5.0	5.0
***All Males***	**37.7 ± 4.4**	77.4 ± 6.5*	76.6 ± 6.3*	**22.3 ± 1.3**	**16.2 ± 2.1**	**22.7 ± 1.6**	**22.5 ± 1.6**	70.8 ± 5.5*	72.0 ± 5.4*	6.6 ± 2.6*	4.8 ± 2.4*	8.5 ± 3.0*	6.2 ± 2.5*
***Combined***	**39.0 ± 5.1**	**73.1±8.8**	**72.3 ± 8.5**	**21.9 ± 1.9**	**15.8 ± 1.9**	**22.8 ± 1.6**	**22.6 ± 1.6**	**62.2 ± 62.9**	**64.8 ± 11.0**	**10.1 ± 5.3**	**7.9 ± 5.7#**	**14.4± 8.2**	**11.0 ± 7.8**

*Denotes significant difference between females and and males (P<0.05). “X” in Female 2 reflects missing data.

### 3.3 Pack weights

Pre- and post-expedition absolute pack weights were not different between females and males (*p* = 0.26), and body weight/pack weight was not different between females and males (*p* = 0.12).

### 3.4 Energy metabolism

Estimated BMR was higher in males compared to females (*p* < 0.0001) (Table 3). EEA (MJ/24 hr) and TEE (MJ/ hr) were greater in males compared to females (*p* = 0.006 and *p* = 0.002, respectively) (Table 3). Physical Activity Level (PAL) trended higher in males than females (*p* = 0.08), but TEE (MJ/FFM/24 hr) was not different between females or males (*p* = 0.28) (Table 3). The sum total of TEE was higher in males (227 ± 52 MJ/expedition) compared to females (170 ± 56 MJ/expedition), but the sum total of TEE relative to FFM was not different between males (3.4 ± 0.7 MJ/FFM/expedition) and females (3.4 ± 1.1 MJ/FFM/expedition).

**TABLE 3:** Energy metabolism female, male, and combined data are presented as mean ± standard deviation.

**Energy Metabolism**	**Basal Metabolic Rate (MJ/24 hr)**	**Exercise Expenditure Activity (MJ/24 hr)**	**Total Energy Expenditure (MJ/24 hr)**	**Total Energy Expenditure (MJ/FFM/24 hr)**	**Physical Activity Level (MJ/BMR)**	**Days in Event**
*Female 1*	5.7	16.4	22.1	0.48	3.9	10
*Female 2*	5.5	11.1	16.7	0.38	3.0	7
*Female 3*	6.6	15.1	21.7	0.39	3.3	9
*Female 4*	5.9	13.5	19.4	0.40	3.3	9
*Female 5*	6.3	21.4	27.8	0.52	4.4	9
*Female 6*	5.8	15.4	21.2	0.45	3.6	9
*Female 7*	7.2	17.6	24.8	0.40	3.5	5
*Female 8*	5.3	7.5	12.8	0.31	2.4	7
**All Females**	**6.1 ± 0.1**	**14.8 ± 4.2**	**20.8 ± 4.7**	**0.42 ± 0.07**	**3.4 ± 0.6**	**8.1 ± 1.6**
*Male 1*	7.8	23.5	31.3	0.46	4.0	10
*Male 2*	7.1	18.2	25.3	0.41	3.6	7
*Male 3*	6.7	22.3	29.0	0.51	4.3	7
*Male 4*	7.4	22.1	29.5	0.45	4.0	7
*Male 5*	7.8	24.7	32.5	0.47	4.2	7
*Male 6*	8.5	36.4	44.9	0.59	5.3	7
*Male 7*	6.8	16.3	23.1	0.39	3.4	8
*Male 8*	8.1	21.2	29.3	0.40	3.6	8
*Male 9*	8.6	19.7	28.3	0.36	3.3	7
*Male 10*	7.1	14,0	21.1	0.34	3.0	7
*Male 11*	7.6	21.1	30.2	0.45	4.0	8
*Male 12*	7.3	32.4	32.4	0.62	5.4	7
**All Males**	7.6 ± 0.6*	22.7 ± 6.3*	31.0 ± 7.5*	**0.45 ± 0.10**	**4.0 ± 0.9#**	**7.5 ± 0.9**
***Combined Females and Males***	**7.0 ± 0.9**	**19.6 ± 6.6**	**26.5 ± 7.3**	**0.44 ± 0.08**	**3.8 ± 0.7**	**7.5 ± 1.7**

*Denotes significant difference between females and males. #Denotes trend towards a difference between females and males. Days in event reflects the number of days for each athlete in the AMWSC.

**FIGURE 2:**
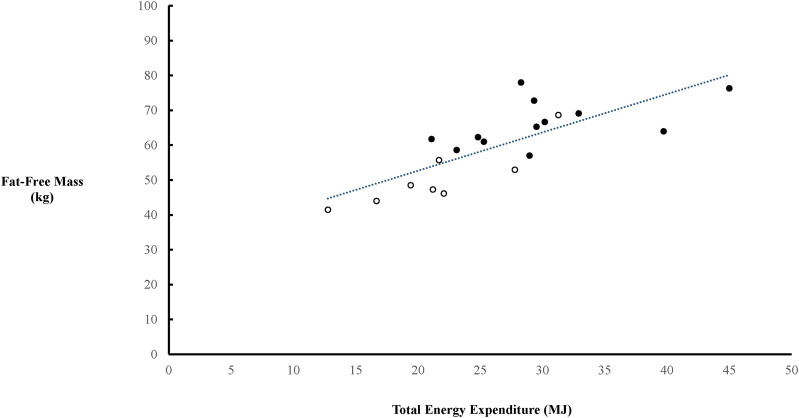
Relationship between fat-free mass (FFM) and total energy expenditure (TEE). The trendline indicated a significant relationship between FFM and TEE in the combined cohort of females (○) and males (●) *(r = 0.7720, p = 0.0004)*. There was also a significant relationship between FFM and TEE in females (r = 0.7440, p = 0.03), but only a trend in males (r = 0.4988, p = 0.0987).

**FIGURE 3:**
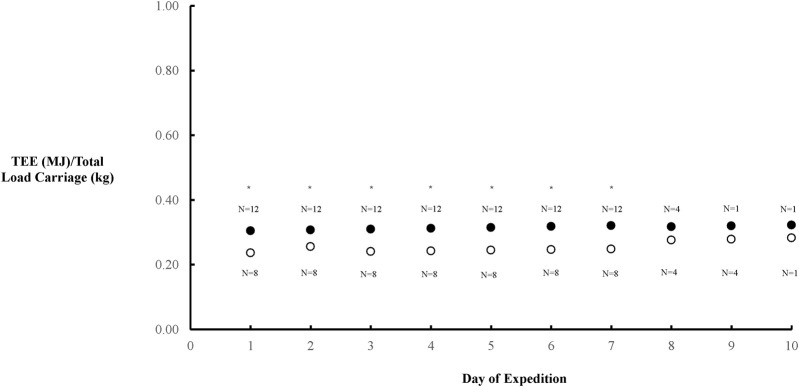
Overall TEE/total load carriage throughout the AMWSC expenditure that was significantly lower in males (●) and females (○) (P = 0.0011). The number of participants for each data point during the event are provided either above or below for females and males, respectively. * Denotes significant difference.

There was a positive relationship between FFM (derived from bioelectrical impedance) and TEE in the combined cohort of females and males (*p* = 0.0004), which continued in females (*p* = 0.03) but only detailed a strong trend in males (*p* = 0.098) (Figure 2). TEE/Total Load Carriage was lower in females compared to males (*p* = 0.0011) (Figure 3).

### 3.5 Water turnover

TBW was significantly lower in females compared to males (*p* < 0.0001). WT was expressed as kg/day, mL/kg/day, or L/MJ. There were no differences in these variables between males and females (Table 4).

**TABLE 4:** Water turnover female, male, and combined data are presented as mean ± standard deviation.

Water Turnover	Total Body Water (kg)	rH_2_O (kg/24 hr)	rH_2_O (ml/kg/24 hr)	rH_2_O (L/MJ)
*Female 1*	34	3.2	50	0.14
*Female 2*	32	5.0	80	0.30
*Female 3*	41	7.3	110	0.33
*Female 4*	35	3.2	50	0.17
*Female 5*	39	4.7	68	0.17
*Female 6*	35	5.6	92	0.27
*Female 7*	45	4.7	55	0.19
*Female 8*	30	5.6	94	0.44
**All Females**	**37 ± 5**	**4.9 ± 1.3**	**75 ± 23**	**0.25 ± 0.10**
*Male 1*	50	4.5	55	0.14
*Male 2*	45	3.3	46	0.13
*Male 3*	42	4.3	60	0.15
*Male 4*	48	5.9	81	0.20
*Male 5*	51	4.9	59	0.15
*Male 6*	56	5.3	62	0.12
*Male 7*	53	6.2	84	0.27
*Male 8*	53	6.7	77	0.23
*Male 9*	57	6.5	75	0.23
*Male 10*	45	4.2	57	0.20
*Male 11*	49	4.9	66	0.16
*Male 12*	47	7.3	103	0.18
**All Males**	49 ± 5*	**5.3 ± 1.2**	**69 ± 16**	**0.18 ± 0.05#**
***Combined Females and Males***	**43 ± 8**	**5.2 ± 1.2**	**71 ± 18**	**0.21 ± 0.08**

*Denotes significant difference between females and males.

#Denotes a trend towards a difference between females and males

## 4 Discussion

The primary aim of the current study was to compare rates of TEE between females and males during the AMWSC expedition in the Arctic winter with temperatures ranging from −36 to −6°C. FFM, BMR (MJ/day), EEA (MJ/day), TEE (MJ/day), and TEE (MJ/Expedition) were higher in males compared to females. However, TEE expressed per FFM was similar in females and males despite differences in absolute TEE (MJ/day), supporting our overarching hypothesis. By comparison, the absolute levels of TEE reported in this Arctic winter expedition represent some of the highest ever reported, even when compared to a wide range of other cohorts from active occupational and recreational communities (Cooper et al., 2011). In the current study, there were minimal changes in body composition in spite of the complex interactions between social isolation, self-sustainability, negative energy balance, environmental challenges, and physiological stressors. Though other cohorts exhibit considerably lower levels of TEE, WT was much less in the current study by comparison to other events or occupational stressors (Ruby et al., 2002). The competitive culture, environmental conditions, external support, exercise intensity, and duration of physical activity differ substantially between these types of events. Nevertheless, these data further demonstrate that even in Arctic conditions, energy balance can be maintained when the TEE approaches the 4x BMR threshold (Cooper et al., 2011).

Almost 40 years ago, Westerkerp et al. described sustained TEE of ∼29 MJ/day in the 1984 Tour de France cyclists (Westerkerp et al., 1986). Twenty-seven years later, the absolute limits of TEE in humans over several days were reviewed extensively. Only five of twenty-five studies employing the DLW method for extended multi-day events reported sustained energy expenditure exceeding 30 MJ/day (Cooper et al., 2011). These studies included elite male cyclists competing in the Giro d’Italia (24-day professional cycling event) and elite Swedish male cross-country skiers who averaged ∼32 MJ/day and ∼30 MJ/day, respectively (Plasqui et al., 2019; Sjödin et al., 1994). Absolute rates of TEE in elite Swedish cross-country female skiers averaged ∼20 MJ/day. The rates of TEE reported in female and male athletes competing in the AMWSC met or exceeded some of the highest rates of DLW-derived TEE ever recorded in humans (Cooper et al., 2011). This is particularly interesting when considering that the AMWSC takes place in an unscripted and unsupported format with no broken trails or pre-established routes provided, all while immersed in an extremely cold and vast wilderness where the margins of survival can be small. By comparison, DLW-derived measurements of TEE in Alaskan sled dogs approach 47 MJ/day (Hinchcliff et al., 1997), highlighting very important species differences in oxygen delivery and utilization, ultimately limiting rates of TEE in humans (Poole and Erickson, 2011).

Acute cold exposure in humans has been demonstrated to augment resting metabolic rates to generate heat (Nakamura and Morrison, 2008). Elegant studies have described cold-induced activation of brown fat, stimulating a great deal of enthusiasm in the literature devoted to the prevention and treatment of metabolic disease (Cohen and Spiegelman, 2015). As part of our ongoing efforts to study metabolic responses during ultra-endurance events in the extreme cold, we previously noted modest alterations in irisin, which is thought to augment brown fat activation (Coker et al., 2017). It is well understood that cold exposure may influence resting metabolism. Nevertheless, these adaptive responses may not be as profoundly affected when heat generation is manifested through relatively low but constant levels of physical activity and retained by breathable, insulated clothing (Haman et al., 2006). More research is needed in unscripted Arctic field environments to more completely understand the impact of extreme cold on metabolism.

It is not surprising that BMR, EEA, and TEE expressed in absolute terms were higher in males compared to females. When TEE is expressed relative to FFM, these differences are diminished with an almost indistinguishable variation between the sexes. There was a positive trend to support a potential difference in PAL between females and males in the current study. This was largely influenced by two male participants who exhibited 5.4 PAL as opposed to an average 3.6 PAL for the remaining male participants. Through our post-expedition conversations with the ASWSC athletes, we discovered that these two male participants literally “broke trail” continuously in deep, sub-par snow conditions, moving over a span of 60 h without sleep. This sustained bout of physical activity influenced their overall values, explaining the disparity from the rest of the group. Expressed as PAL or TEE (MJ/FFM/day) or TEE (MJ/FFM/expedition), there was no difference between females and males, which demonstrates the influence of FFM on TEE and remains consistent with many observations in a wide variety of circumstances (Coker et al., 2021; Ruby et al., 2002; Pontzer et al., 2021; Hunter 2016).

Remarkably, TEE/Total Load Carriage over the course of the expedition was lower in females compared to males. Our observation is congruent with those that show women to be potentially more resistant to performance fatigability (Glace et al., 2013). Our previous study in this same cohort demonstrated that while women carried more pack weight/body weight, their physical performance was not negatively affected by this discrepancy (Johannsen et al., 2018). Prior studies have suggested more resistance to peripheral fatigue in females compared to males, potentially through higher dependency on lipids as a relatively inexhaustible fuel substrate (Hunter 2016). This is especially so in women who may have greater mitochondrial net oxidative capacity, as recently demonstrated (Giuriato et al., 2024). For example, six females dragging 80 kg gear sleds were comprehensively evaluated during a 61-day Antarctic Traverse. The estimated average TEI was 20.8 ± 0.1 MJ/day, and these athletes lost ∼13% of their total body mass, or 9.4 ± 2.3 kg (Gifford et al., 2019). Despite the extreme nature of the expedition and the pronounced negative energy balance, FFM was preserved and the pituitary gonadotrophic reactivity was not impaired. This data provides a unique example of physiological resilience despite the extreme conditions and negative energy balance. Unscripted studies under austere circumstances are uncommon compared to laboratory-based investigations, and females have been woefully underrepresented in this regard. Therefore, additional studies are needed to gain a further understanding of the sex-specific mechanisms responsible for fatigability during exercise (Hunter 2016).

We have repeatedly demonstrated the preservation of FFM, lean tissue mass, and/or skeletal muscle during relatively constant physical activity in humans under conditions of negative energy balance (Johannsen et al., 2018; Coker et al., 2018; Coker et al., 2017; Ruby et al., 2002). Even in the Talisker Whiskey Atlantic Challenge, which lasts ∼46 days, skeletal muscle was largely preserved as measured by ultrasound (Holsgrove-West et al., 2024). On the other hand, US Army Ranger training combines prolonged periods of negative energy balance (i.e., 2-month minimum) with continuous physical activity and negligible energy intake. These stresses exist in conjunction with sleep deprivation and high load carriage, leading to significant reductions in FFM and physical performance (Nindl et al., 2007). Once the total negative energy balance reaches ∼167 MJ, the resiliency of skeletal muscle retention seems to wane. As a result, physical performance declines, and the risk of injury increases (Murphy et al., 2018). To put negative energy balance into perspective, it would require 10 days at a negative energy balance of 16.7 MJ or 3,989 kcal, or 20 days at 8.4 MJ or 2,006 kcal to result in muscle loss and/or negative impacts on physical performance. While this threshold is less plausible for individuals participating in relatively short-term events, functional capability is more likely to be negatively affected by longer events such as sustained military field training exercises.

WT was ∼30–50% lower in the current cohort compared to other athletes and occupational circumstances in warmer environments (Ruby et al., 2003; Rosales et al., 2021; Nelson et al., 1947). Arctic winter conditions are devoid of heat and humidity, which may reduce the need to replace fluids lost to high sweat rates in hot and humid environments (Murray, 2022). While very important to overall sustainability, securing drinkable water in an extremely cold and completely unsupported environment is challenging. Under such conditions, water in its liquid form is difficult to locate. When found, it presents a potentially dangerous situation for retrieval under likely overflow ice or “aufeis” conditions. Available water is likely viscous and will cause filtration systems to malfunction or fracture. Boiling water requires dexterity to access and manipulate cook kits, exposing extremities to the risk of frostbite. Therefore, the complexities of water procurement in the Arctic environment affect WT.

In these environmental circumstances, humans must strike a fine balance between sufficient physical activity and adequate clothing to keep them relatively “warm.” This balance falls short of exercise intensities and/or insulated barriers that result in excessive sweating which would allow the moisture to freeze, therefore exposing the athletes to a high risk of hypothermia (Airmen, 2022). For instance, metabolic heat production increases 5-fold, from 210 W at light physical activity to 1,048 W at very high to intense maximal work (Ainsworth et al., 2000). It is difficult to ascertain or compare relative work rates or exercise intensity using the DLW method in field studies, especially those that are unscripted and unsupported. However, one could certainly surmise that athletes would prefer reaching or maintaining a work rate below undesirable sweat rates, a preference which is not necessarily relevant or even important in supported events occurring in thermoneutral or hot environments.

There were a few important limitations to our study. We did not measure any sleep related parameters in this study. From our conversations with the athletes, it seems that they get no more than 4–5 h/night, of which the sleep quality is likely to be negatively affected by the extremely cold temperatures. Given that impairments in the amount and quality of sleep negatively affect recovery through increased catabolic and decreased anabolic hormones (Fullagar and Bartlett., 2016), the event is all the more challenging. Future studies are planned to assess the impact of sleep deprivation on the muscle proteome under similar conditions. Also, we did not measure skin temperature or core temperature of the athletes, which may have been affected by minor variations in clothing strategies, *etc.* Previous studies have demonstrated that insulation albeit *via* body fat does not affect metabolic rate under modest cold conditions (Glickman-Weiss et al., 1999), but it is not clear whether these observations are consistent under circumstances of prolonged exposure to extreme cold. Nevertheless, similar strong relationships between fat-free mass and TEE persist. Lastly, we did not control for or evaluate the menstrual status of our female participants due to the inherent nature of the unscripted event itself and the potential for additional stress related to blood sampling, etc*.* Menstrual status has been suggested to influence the anabolic hormonal profile, but some elegant laboratory based studies have refuted this assertion (Colenso-Semple, 2024). Nevertheless, we are currently conducting additional studies in somewhat similar circumstances where we are assessing menstrual status and potential relationships to muscle metabolism.

## 5 Conclusion

We have demonstrated high rates of TEE in female and male athletes participating in the AMWSC, a self-supported, remote Arctic winter expedition in the Brooks Range of Alaska. While there were absolute differences in TEE between females and males, these variations were normalized when expressed relative to FFM. Lower TEE relative to total load carriage in females compared to males may cautiously suggest greater resistance to fatigue in females under these conditions. Our observations regarding the potential for improved metabolic efficiency in females should be viewed with caution due to the use of data imputation and bioelectrical impedance (McCleary 2002). Not unlike other studies describing similar rates of TEE over relatively short time frames, FFM was preserved. By descriptive comparison to previous studies, WT was lower (5.2 ± 1.2 L/day) despite TEE of ∼30 MJ/day. This observation may be potentially due to the management of exercise intensity by the AMWSC athletes. Maintaining lower exercise intensity allows athletes to mitigate excessive sweat rates in the austere environmental conditions of an Arctic alpine winter expedition. Finally, males and females presented similarities in TEE and WF when expressed relative to FFM. The extreme Arctic winter conditions, coupled with the physical demands of the AMWSC, present physiological stressors that yield high rates of TEE in females and males, while lower WT is noted. Lastly, we did not control for menstrual phase of the female participants as such an approach would have burdensome to these athletes and drastically limited participation.

## Data Availability

The original contributions presented in the study are included in the article/supplementary material, further inquiries can be directed to the corresponding author.
